# Case of Hiccough Successfully Treated by Inhalation of Amyl Nitrite

**Published:** 1875-12

**Authors:** C. Paul Simon

**Affiliations:** Chicago


### A CASE OF HICCOUGH SUCCESSFULLY TREATED BY INHALATION OF AMYL NITRITE.

By Dr. C. PAUL SIMON, Chicago.

[Reported to the Chicago Society of Physicians and Surgeons.]

The patient was a fireman, aet. 35 years, who had hiccoughed for twenty-six hours, and had been subjected to various treatments without success. Remembering the use of the amyl nitrite, so far back as 1867, in angina pectoris, by Lander Brunton, the reporter was led to administer it in this case, by reason of the analogy of these two nervous affections. Three drops of the amyl were inhaled, and the hiccough stopped instantaneously. All authors agree as to the ultimate effects of this drug, to wit: a diminution of the arterial pressure, and consequently a dilatation of the blood vessels. Schüller (vide Virchow's *Jahresbuch für* 1870) showed this diminished pressure and vascular dilatation on rabbits whose skulls had been trepanned. The primary effect of the drug is either its impression on the blood (Ladendorf, l. c.), its paralyzation of the peripheral vaso motor nerves (Lander Brunton), or its checking the excitability of the vaso motor centres (Felehore, l. c.); which action it exerts is not yet satisfactorily settled. Section of either pneumogastric or of the medulla oblongata, is followed by dilatation of the little vessels of the pia mater and by pulsation of the veins (Schüller, l. c.). The dilatation obtained by the tearing off of the sympathetic ganglion of the neck can be increased by inhalation of amyl nitrite. From the evident success of treating all cases of anæmia of the central nervous organs, it follows in this particular case that the hyperkinesia of the phrenic nerve might be due to an anæmia of the upper part of the medulla.

Oct. 11, 1875.

